# A Systematic Review of Food Deserts, 1966-2007

**Published:** 2009-06-15

**Authors:** Julie Beaulac, Elizabeth Kristjansson, Steven Cummins

**Affiliations:** St Boniface General Hospital. At the time of this research, Ms Beaulac was affiliated with the University of Ottawa, Ottawa, Ontario, Canada.; University of Ottawa, Ottawa, Ontario, Canada; University of London, London, England

## Abstract

**Introduction:**

"Food deserts," areas characterized by poor access to healthy and affordable food, may contribute to social and spatial disparities in diet and diet-related health outcomes. However, the extent to which food deserts exist is debated. We review the evidence for the existence of food deserts in socioeconomically disadvantaged areas.

**Methods:**

We conducted a systematic review of primary, quantitative, observational studies, published in English or French, that used geographic or market-basket approaches in high-income countries. The literature search included electronic and hand searches and peer-reviewed and grey literature from 1966 through 2007. We also contacted key researchers to identify other studies. We analyzed the findings and quality of the studies qualitatively.

**Results:**

Forty-nine studies in 5 countries met inclusion criteria; the amount and consistency of the evidence varied by country. These studies were a mix of geographic and market-basket approaches, but the methodologic quality of studies and completeness of reported findings were mixed. We found clear evidence for disparities in food access in the United States by income and race. Findings from other high-income countries were sparse and equivocal.

**Conclusion:**

This review suggests that food deserts exist in the United States, where area-level deprivation compounds individual disadvantage. Evidence for the existence of food deserts in other high-income nations is weak.

## Introduction

"Food deserts," areas characterized by relatively poor access to healthy and affordable food, may contribute to social disparities in diet and diet-related health outcomes, such as cardiovascular disease and obesity (1-3). The term "food desert" reportedly originated in Scotland in the early 1990s and was used to describe poor access to an affordable and healthy diet (4). Although the term "food desert" can mean a literal absence of retail food in a defined area, studies of food deserts more commonly assess differential accessibility to healthy and affordable food between socioeconomically advantaged and disadvantaged areas.

In the last decade, research on food deserts has become increasingly common, but this research is also a source of debate (4). Many primary studies have been conducted on food deserts, and some attempts have been made to compile the existing evidence (2,5-7). Despite the burgeoning interest in food deserts, we could find no systematic review of them. We address this gap by systematically reviewing the evidence for food deserts, specifically in socioeconomically disadvantaged areas. Food deserts in disadvantaged areas are arguably of more concern because a process of "deprivation amplification" may contribute to social disparities, whereby area-level deprivation compounds individual disadvantage (8,9). The objective of this review was to systematically and critically review the literature to determine whether access to healthy, affordable food in retail stores varies by area socioeconomic status to the disadvantage of socioeconomically deprived areas. In addition, we critique the methodologic rigor of existing evidence.

## Methods

We included quantitative studies from high-income countries (10) if they compared 2 or more geographic areas that differed by socioeconomic indicator of disadvantage and, for market-basket comparisons, involved more than 1 type of food. We considered market-basket studies that compared a selection of food items across areas and stores in terms of availability, variety, price, or quality; geographic studies that compared the accessibility of different types of food stores; and studies that combined geographic and market-basket approaches. We allowed both random and nonrandom sampling methods; however, we excluded studies that compared preidentified food deserts to other areas. We also excluded studies that investigated change as a function of time or intervention and studies that did not involve food retail stores (eg, restaurants) (11,12). Eligible outcomes included average distance to nearest food stores, store density by area or population, average selling space, availability and variety of items, price of food, and food quality. Variety was defined as number of different types or brands of the same food item.

We searched the following electronic databases for all years since inception of the database through September 2007: CINAHL, EMBASE, MEDLINE, PsycINFO, Social Services Abstracts, Sociological Abstracts, and ABI INFORM/Global. We searched these databases for original research articles written in English or French. The OVID search strategy used free-text terms and subject headings (Appendix A). We searched other databases and the *Journal of Business* online by using relevant free-text terms and conducted a grey literature search by using Google and Google Scholar. We hand-searched reference lists of included studies and contacted key researchers in the field (Diez-Roux, Giskes, Raine, Turrell, and Zenk) to identify other relevant studies.

Two authors independently reviewed search results, reviewed retrieved studies to determine whether they should be included, and abstracted outcomes data. We independently assessed the methodologic quality of studies that met our inclusion criteria. Instead of rating or scoring each study, we rated the quality to describe how methodologically robust each included study was. Six criteria were common to all types of studies, 3 were unique to market-basket studies, and 1 was unique to geographic studies (Appendix B). When findings were reported in several articles, we used the most recent published version to rate quality and outcomes, unless different outcomes were published in different articles (13,14).

## Results

The literature search identified 2,826 studies; of these, 106 were retrieved. After detailed inspection of retrieved studies, 57 were excluded and 49 were included. Reasons for exclusion were irrelevant outcomes or comparisons (n = 21), not a study (n = 6), inappropriate method of gathering or using data (n = 2), methods not relevant (n = 16), inappropriate or no comparison area (n = 15), only 1 food item studied (n = 1), inadequate information (n = 10), and preselected food deserts (n = 3). Some studies were excluded for multiple reasons.

The 49 included studies consisted of 22 market-basket studies, 17 geographic studies, and 10 mixed-methods studies (Appendix C). Thirty-seven were from published sources, and 12 were from unpublished sources. Thirty-four were conducted in the United States, 5 in the United Kingdom, 6 in Canada, 3 in Australia, and 1 in New Zealand. They varied in scope from a comparison of 2 areas in 1 city (15) to a comparison of food stores across the United States (16). Dates ranged from 1966 to 2007; 3 studies were published in the 1960s, 2 in the 1970s, 2 in the 1980s, 12 in the 1990s, and 29 after 2000; 1 study had no date. Some geographic studies considered all types of food retail stores, whereas others only considered supermarkets.

Among market-basket studies, the number of food items in baskets varied; the largest basket included more than 80 items (17) and the smallest included 18 items (18). Some studies focused on 1 type of basket (eg, typical market basket or a healthy food basket), and others compared a healthy food basket to an unhealthy food basket.

The 17 geographic survey studies were of moderately high quality. Reporting of raw data and statistics and precision of data were problematic in some studies. The 10 mixed-methods studies were higher quality; however, nearly all failed to report interrater reliability. The quality of the 22 market-basket studies was variable and generally poorer. Interrater reliability was rarely reported; many also had problems with store selection, reporting raw data, statistics, and participation rate. In addition, many of these studies did not control for item quality and only compared outcomes within type of store and not across neighborhood.

### Geographic survey outcomes

**United States**

Geographic areas with a high proportion of low-income or African American residents were underserved by food retailers compared with more advantaged areas (19 studies: 18 in support, 1 mixed). Evidence on rural/urban differences was mixed.

Low-income areas (8 studies) and areas with a high proportion of African Americans (5 of the 8 studies) had fewer supermarkets or chain stores per capita (3,7,14,19-22) and fewer midsized or large stores (23) than did advantaged areas. Three studies combined income and race; in these, areas characterized by low income and a high proportion of African Americans and Latinos had few supermarkets or chain stores per capita (24-26). Four studies that were unadjusted for population found few supermarkets or chain stores in low-income (5,27-29) and African American areas (5).

Distance to supermarkets (3,13,29) was farther for those living in low-income areas and for areas with a high proportion of African Americans (3,30). In addition, 2 studies found that supermarkets in low-income areas had less selling space (21,23). In another study (31), stores in middle-income neighborhoods had more selling space than did those in low- and high-income neighborhoods. Three studies found that low-income areas with a high proportion of African Americans had more convenience stores (22,24,25), while 1 study found no difference (29).

When comparing rural and urban areas, rural areas had less store selling space (31). In contrast, 1 small study found that 2 rural areas had more grocery stores per capita than did 2 urban areas (32).

**United Kingdom**

Low-income areas had fewer chains but more large, independent stores and greengrocers per capita (33). One study found more food stores of all types in deprived areas compared with more advantaged areas (34).

**Canada**

One of 3 Canadian studies showed half as many grocery stores and 3 times more convenience stores in a low-income area (15). One study (35,36) found mixed results; distance to stores was shortest for middle-income areas. In another, low-income areas were better served by stores than other areas (37).

**Australia and New Zealand**

The relationship between area income and store accessibility was nonlinear in 1 Australian study (38), such that middle-income areas had the most stores, but low-income areas were closer to a supermarket. Rural New Zealanders (39) and Australians (40) lived farther from stores than did urban residents. In addition, rural areas in Australia had fewer stores per capita (40).

### Market-basket outcomes

**United States**

Findings regarding price were mixed and complex. One study showed higher prices in low-income areas (20), and another (41) found higher prices for African American areas but mixed results by area income. Six studies (18,42-46) had mixed results by area income (eg, lower prices in middle-income areas than in lower- and higher-income areas or similar prices overall but higher prices on items of comparable quality); another was mixed for area income and race together (17). In addition, 4 studies (28,47-49) showed no difference in price, and 3 found that prices were lower in low-income or African American areas (24,25,50). Prices were lower in urban than in rural areas (47,51,52), while 1 study reported mixed results (32).

In terms of availability and quality of healthy foods, disadvantaged areas fared poorer; 5 studies found poorer accessibility for disadvantaged areas (19,25,26,45,50), and 4 found mixed results (24,41,49,53).

**United Kingdom**

Findings regarding price were variable. One study found slightly higher prices for healthy food in low-income areas (54), while another found differences only in fruit and vegetable prices (33). Other studies found few differences (55) and lower prices for healthy food in low-income neighborhoods than in high-income neighborhoods (56).

Two studies found lower availability (54,56) and lower variety of healthy foods (56) in disadvantaged areas. Two other studies (33,55), however, found no difference in availability. One study found food quality to be worse in low-income areas (54), although another reported no difference (33).

**Canada**

Food availability (15,57) and prices (15,58) did not differ significantly between low- and high-income areas. However, rural and remote areas had higher prices (58,59) and poorer availability for all types of food baskets (58).

**Australia and New Zealand**

No differences were found between low- and high-income areas regarding availability and variety, and differences in price were mixed (60). In another study, availability of food decreased and the price of a healthy food basket increased steadily as area remoteness increased (61).

## Discussion

Food deserts exist, at least in the United States. Evidence is both abundant and robust enough for us to conclude that Americans living in low-income and minority areas tend to have poor access to healthy food. However, studies on the price of food were generally of low quality, and their findings were mixed. Evidence from other countries is sparse and equivocal. The evidence that is available is much less compelling than evidence from the United States. On this basis, evidence from other countries does not warrant firm conclusions at this time on whether access to healthy, affordable food systematically varies to the disadvantage of socioeconomically deprived areas.

The environment in which we live, work, and play contributes to health and socioeconomic differences in health over and above the influence of individual characteristics (62). We found evidence for structural inequalities in the food retail environment and believe that these inequalities may contribute to inequalities in diet and diet-related outcomes. For the United States, our findings suggest a process of deprivation amplification (9), since structural problems related to food retail appear to further disadvantage low-income and minority Americans, who are already limited in their ability to purchase healthy food.

Less access to supermarkets and chain stores in low-income neighborhoods may create barriers to accessing healthy, low-cost food for those who lack access to transportation; in 2001, 26.5% of Americans with incomes below $20,000 did not own a car (63). This barrier, combined with the increased presence of small, independent stores and convenience stores in low-income neighborhoods, may limit shopping to stores that charge higher prices (24,42). These stores have a poor selection of healthy foods and a wide selection of unhealthy foods, which can contribute to poor diet. In the United States, increased access to supermarkets is associated with lower prevalence of overweight and obesity (64), improved fruit and vegetable consumption, and better diet quality among African Americans (13,65), low-income households (66), and pregnant women (67). In contrast, increased access to convenience stores is associated with increased risk of obesity (64).

In considering the influence of the environment on health, the quality of existing amenities must be taken into account (9), in addition to their availability and accessibility. This review shows that low-income areas are also disadvantaged in this regard. Availability of healthy food is associated with better diet (68); perception of better selection and quality of fresh fruit and vegetables is associated with increased fruit and vegetable consumption (65).

Evidence on neighborhood food price from the United States was inconsistent. However, studies showed higher prices among convenience stores and small, independent stores, which are more prevalent in low-income and African American communities. Furthermore, the finding of higher prices for healthier food baskets (45) is supported by a recent economic analysis (69) that demonstrated that energy-dense diets cost less than healthier diets. These observations suggest that people with limited food budgets may not be able to purchase healthy food. Indeed, people on low incomes cite the high cost of healthy foods as a barrier to eating healthily (45,70). Lower fruit and vegetable prices are associated with lower body mass index among elementary school children; effects are particularly strong for those children who are disadvantaged by poverty or race (71).

### Implications for policy

A recent article (9) highlighted the need for nutrition policy to be based on sound research evidence, but the government of the United Kingdom has been pursuing a policy agenda based on evidence from early, small studies (4). Our review concurs with the conclusion that there is little evidence that socioeconomically deprived areas in the United Kingdom are systematically disadvantaged by food deserts. We do not mean to imply that accessing food in the United Kingdom is without challenges, but more evidence is needed.

In contrast to the situation in the United Kingdom, evidence shows that socioeconomic inequalities in nutrition environments exist in the United States. Therefore, plausible and equitable policy and planning responses are needed. We suggest that local, federal, and state governments consider environmental and social interventions to decrease price disparities between healthy and unhealthy foods, facilitate the entry of supermarkets and other food stores into low-income areas, encourage the development of local grocery cooperatives, encourage the advertisement of healthy foods, and foster the development of more community food projects.

This review illustrates the salience of context; evidence is not necessarily generalizable across countries or even between municipalities in some countries. The fact that evidence from different countries varies widely suggests national and regional differences in planning regulation and law, patterns of residential segregation, and differing local social and cultural environments (9,72). The conclusions of this review may change after the evidence base is expanded for countries where evidence is sparse.

### Implications for research

Further research on this topic is warranted, particularly in countries other than the United States. Studies should incorporate both market-basket and geographic surveys. We recommend random samples or censuses of all stores in study areas. Before sampling, all food retail outlets in an area (including convenience stores) should be characterized and classified, and prespecified boundaries should be strictly adhered to. Thoughtful consideration should be given to market baskets that reflect local tastes and preferences. Market baskets should also contain discriminator items, such as healthy, high-quality foods, rather than just including foods for a low-income diet. In addition, prices of healthy and unhealthy food should be contrasted (73). Food and store quality should be rated, and prices of items of similar quality and size should be compared.

Although the studies included in this review improve our understanding of disparities in access to health-promoting resources, future studies should be extended to routinely include data on residents' shopping and health behaviors and health outcomes (1). When such data are included, we can fully quantify the effect of socioenvironmental risk on diet and related diseases.

## Appendices

### Appendix A: OVID Search Strategy for a Systematic Review of Food Deserts, 1966-2007

Electronic databases searched:

CINAHL (from 1982)

EMBASE (from 1980)

MEDLINE (from 1966)

PsycINFO (from 1806)

Social Services Abstracts (from 1979)

Sociological Abstracts (from 1952)

ABI INFORM/Global (from 1971)

1. ((food or foods or grocer$) adj3 (available or availability or affordability or affordable or price or pricing or prices or cost or costs or retail$ or store or stores or supermarket$ or market or markets or shop or shops)).ab,ti.
2. food supply/
3. (food adj3 outlet$).ab,ti.
4. or/27-29
5. exp *Residence Characteristics/
6. exp *community/
7. exp *Socioeconomics/ or exp *Socioeconomic Factors/ or exp *sociocultural factors/ or exp *socioeconomic status/
8. (neighbourhood$ or neighborhood$).ab,ti.
9. or/31-34
10. (deprived or deprivation).ab,ti.
11. (poor or poorer or poverty).ab,ti.
12. (disadvantaged or disadvantage).ab,ti.
13. 36 or 37 or 38
14. 35 or 39
15. 30 and 40
16. ((food or foods or shopping) adj (desert or deserts)).ab,ti.
17. 41 or 42

### Appendix B: Criteria for Rating Study Methodologic Quality of Articles Considered for a Systematic Review of Food Deserts, 1966-2007

**All Studies**

1. Was (were) the hypothesis/question (or hypotheses/questions) clearly specified?

- Adequate: consistent and clearly specified research hypothesis/question (or hypotheses/questions)
- Unclear: not clearly specified
- Inadequate: inconsistent or unclearly specified research hypothesis/question (or hypotheses/questions)

2. Were the study areas clearly defined?

- Adequate: systematic and clearly defined geographic neighborhoods, definition consistent across areas
- Unclear: not clearly specified
- Inadequate: poorly defined geographic neighborhoods; inconsistent definition of neighborhood

3. Was the definition of disadvantage consistent and clear?

- Adequate: consistent and clear definition of socioeconomic disadvantage; used consistently across areas
- Unclear: not clearly specified
- Inadequate: inconsistent or unclear definition of socioeconomic disadvantage; not used consistently across areas

4. Was the selection of grocery stores adequate?

- Adequate: total coverage or random selection (stratified by type)
- Unclear: not clearly specified
- Inadequate: nonrandom selection

5. Was the reporting of raw data adequate?

- Adequate: raw data are presented and described adequately
- Unclear: not clearly specified
- Inadequate: raw data are not presented or described adequately

6. Was the statistical reporting adequate?

- Adequate: significance and effect size calculated, statistical analyses used
- Unclear: not clearly specified
- Inadequate: significance and effect size not calculated, descriptive or inappropriate analyses used

**Market-Basket Studies Only**

7. Was the participation rate sufficient?

- Adequate: ≥80%
- Unclear: not reported
- Inadequate: <80%

8. Was the reliability of raters adequate?

- Adequate: raters trained, good interrater reliability
- Unclear: unable to judge
- Inadequate: poor interrater reliability

9. Was the reliability of outcome measures, including the composition of the market baskets, adequate?

- Adequate: standard measure used, consistency in brand, size, and quality
- Unclear: unable to judge
- Inadequate: brands, size, quality inconsistent

**Geographic Survey Studies Only**

10. Was the precision of data adequate?

- Adequate: used weighted averages/density
- Unclear: not clearly specified
- Inadequate: used counts

### Appendix C: Studies Included in a Systematic Review of Food Deserts, 1966-2007

**Table:**

Country	Authors
**Market-basket studies**	
Australia	Lee, Darcy, Leonard, Groos, Stubbs, Lowson, et al (61)
Canada	Bertrand (57)
	Lawn, Robbins, Hill (59)
	Travers, Cogdon, McDonald, Wright, Anderson, Maclean (58)
United Kingdom	Cummins, Macintyre (55)
	Mooney (56)
	Sooman, Macintyre, Anderson (54)
United States	Alcaly, Klevorick (42)
	Ambrose (51)
	Andrews, Kantor, Lino, Ripplinger (43)
	Captain, McIntire (44)
	Crockett, Clancy, Bowering (47)
	Glanz, Sallis, Saelens, Frank (50)
	Green (20)
	Hall (41)
	Hayes (48)
	Jetter, Cassady (45)
	MacDonald, Nelson (46)
	Marcus (17)
	National Commission on Food Marketing (18)
	Sallis, Nader, Atkins (53)
	Wright Morton, Oakland, Bitto, Sand (52)
**Geographic survey studies**	
Australia	O'Dwyer, Coveney (40)
Canada	Apparicio, Micic, Shearmur (35) and Apparicio, Cloutier, Shearmur (36)
	Smoyer-Tomic, Spence, Amrhein (37)
New Zealand	Pearce, Witten, Bartie (39)
United Kingdom	Cummins, Macintyre (34)
United States	Alwitt, Donley (27)
	Cotterill, Franklin (21)
	Dalton, Ehrlich, Flores, Heberlein, Niemeyer (5)
	Helling, Sawicki (30)
	Moore, Diez Roux (22)
	Morland, Wing, Diez Roux, Poole (13) and Morland, Wing, Diez Roux (14)
	Morris, Neuhauser, Campbell (29)
	Hartford Food System (31)
	Powell, Slater, Mirtcheva, Boa, Chaloupka (16)
	Shaffer (7)
	The Brookings Institution (23)
	Zenk, Schulz, Israel, James, Bao, Wilson (3)
**Mixed studies**	
Australia	Winkler, Turrell, Patterson (38,60)
Canada	Latham, Moffat (15)
United Kingdom	White, Bunting, Williams, Raybould, Adamson, Mathers (33)
United States	Baker, Schootman, Barnidge, Kelly (19)
	Block, Kouba (24)
	Chung, Myers (28)
	Horowitz, Colson, Hebert, Lancaster (25)
	Sloane, Diamant, Lewis, Yancey, Flynn, Nascimento, et al (26)
	Smith (32)
	Zenk, Schulz, Israel, James, Bao, Wilson (49)
